# Reference conditions and threshold values for nitrate-nitrogen in New Zealand groundwaters

**DOI:** 10.1080/03036758.2023.2221034

**Published:** 2023-06-12

**Authors:** Christopher J. Daughney, Uwe Morgenstern, Magali Moreau, Richard W. McDowell

**Affiliations:** a National Institute of Water and Atmospheric Research, Wellington, New Zealand; b GNS Science, Wellington, New Zealand; c GNS Science, Wairakei, New Zealand; d Faculty of Agriculture and Life Sciences, Lincoln University, Lincoln, New Zealand

**Keywords:** Groundwater quality, reference conditions, nitrate, hierarchical cluster analysis, groundwater age, land-use impact

## Abstract

Management of groundwater quality is assisted by an understanding of reference conditions, which describe the concentration ranges expected for key substances in the absence of human impact. This study evaluates reference conditions for NO_3_–N in New Zealand groundwater based on three complementary methods: hierarchical cluster analysis, relationships to groundwater age, and regression against a measure of land-use impact. The three methods result in very similar national-scale estimates of reference conditions for NO_3_–N concentration in oxic, minimally impacted groundwater, with the 80th, 90th and 95th percentiles found to be 1.65 ± 0.12, 1.97 ± 0.14 and 2.32 ± 0.14 mg/l, respectively (weighted average ± 95% confidence level), in good general agreement with previous studies from New Zealand and overseas. Anoxic groundwaters were treated separately for definition of reference conditions, with the 80th and 90th percentiles of NO_3_–N found to be 0.04 ± 0.01 and 0.16 ± 0.01, respectively (the 95th percentile could not be estimated reliably). For both oxic and anoxic groundwater, where a site-specific investigation has not been conducted to estimate reference conditions at a local scale, we suggest that the 80th percentile is an appropriate national-scale default threshold, to match the thresholds used for surface waters under the Australian and New Zealand Guidelines for Fresh and Marine Water Quality.

## Introduction

Elevated nitrate-nitrogen (NO_3_–N) concentration in groundwater is a worldwide issue (Abascal et al. 2022). Excess NO_3_–N in groundwater is derived from a variety of current and legacy sources including agriculture, wastewater, stormwater and industry (Wang et al. 2013; Abascal et al. 2022). Elevated NO_3_–N concentrations can affect groundwater ecosystems, compromise the use of groundwater as a potable water supply and, through groundwater-surface water interactions, affect the quality of surface water and dependent ecosystems in receiving environments (Carpenter et al. 1998; Marmonier et al. 2018).

In New Zealand, NO_3_–N inputs into freshwater systems are primarily from livestock urine (Parfitt et al. 2012). Urine patches contain 600–1000 kg N ha^−1^ (Monaghan et al. 2007). These loads are well above the nitrogen requirements for pasture growth (*cf*. 200 kg N ha^−1^), which leaves the remainder susceptible to leaching (Di and Cameron 2007). Elevated NO_3_–N concentrations and increasing temporal trends have been reported for several long-term groundwater monitoring sites across New Zealand (Daughney and Wall 2007; Daughney and Randall 2009; Morgenstern and Daughney 2012; Moreau et al. 2016; StatsNZ 2020), a pattern also evident in surface waters (StatsNZ 2022).

Understanding of reference conditions for NO_3_–N in groundwater is vital for selecting appropriate management approaches and objectives (Shand et al. 2007). Reference conditions specify the expected range of NO_3_–N concentrations in groundwater in the absence of human impacts. The terms baseline and background are also used (Edmunds et al. 2003; Reimann et al. 2005), but here we use the term reference conditions due to its common use in freshwater policy (Hess et al. 2020). Understanding of reference conditions enables partitioning of observed NO_3_–N concentration into the fraction that is anthropogenic vs. natural. Without such understanding, it is difficult to quantify the magnitude of human impacts on groundwater quality (Brydie et al. 2014). In practice it can be scientifically challenging to determine reference conditions representative of the complete absence of human impacts and so some studies instead develop estimates for minimally disturbed conditions (Huo et al. 2012; McDowell et al. 2013; Swanson et al. 2020), a convention also taken in the present study. Regardless of whether defined as a complete absence or a minimal level of human disturbance, understanding of reference conditions enables policy-makers, land managers and communities to set limits or targets for NO_3_–N concentrations in groundwater that are realistic and achievable.

Reference conditions are described as a distribution, not a single number, to encapsulate natural range and variation (Reimann et al. 2005; Shand et al. 2007; Edmunds and Shand 2008). Statistically derived numeric thresholds are often selected from the distribution to assist comparison to concentrations that have been measured in water quality investigations. For example, the Australian and New Zealand Guidelines for Fresh and Marine Water Quality define Default Guideline Values for many water quality parameters in surface water, which are based on the 20th and 80th percentiles of the distribution of concentrations inferred to occur under reference conditions (ANZG 2018). Other thresholds have also been recommended, including the 95th percentile (Edmunds et al. 1987) or the 97.7th percentile (Langmuir 1997). The 90th percentile has also been proposed as an appropriate threshold for small datasets (fewer than 60 measurements) or where human impacts cannot be unequivocally excluded (Hinsby et al. 2008). Note that reference condition thresholds are not necessarily management limits or targets that needs to be met, but rather their exceedance may simply be taken as a prompt to consider whether further investigation should be undertaken to determine whether aquatic ecosystems are sufficiently protected.

Reference conditions are often defined separately for specific water bodies or types of water bodies. For example, the range of natural concentrations of NO_3_–N in groundwater may vary according to aquifer lithology, redox conditions, flow-paths (including aquifer depth) and groundwater age. Unless there is a geological source of NO_3_–N, its concentration under reference conditions is likely to be low under anoxic (reducing) conditions where nitrate is removed through microbiologically-facilitated denitrification, especially in older groundwater. In contrast, detectable levels of NO_3_–N are expected for oxic groundwaters, even in the absence of human impacts (Madison and Brunett 1985; Daughney and Reeves 2005). Accordingly, different definitions of reference conditions (and their corresponding percentile-based thresholds) may be needed for different aquifers, parts of aquifers, or aquifer types (Shand et al. 2007).

A variety of methods are available to estimate reference conditions for freshwater quality. Ideally, reference conditions could be determined from measurements made at monitoring sites located in pristine, unimpacted locations (Shand et al. 2007; ANZG 2018). However, this approach is often hampered by a lack of monitoring sites in pristine areas (Dodds and Oakes 2004). This is the case in New Zealand (Daughney et al. 2012; McDowell et al. 2013), especially for groundwater.

In the absence of monitoring sites in pristine locations, another option is to use historical data from any or all available sites regardless of their location, but to estimate reference conditions based only on samples collected in pre-industrial time periods (Edmunds et al. 2003; Limbrick 2003; Griffoen et al. 2008; Hinsby et al. 2008). However, use of historical data may not be possible because older measurements may be of poor quality or not available at all (Shand et al. 2007). As an alternative to reliance on historical measurements, water dating techniques can be used to estimate groundwater quality prior to anthropogenic activity (Morgenstern and Daughney 2012; Huang et al. 2012). Isotope ratios such as ^18^O/^16^O and ^15^N/^14^N can also be used with the aid of modelling to estimate the source of NO_3_–N and a mass balance of N, allowing the estimation of reference conditions for aquifers with mixed inputs (Minet et al. 2017).

In the absence of data from unimpacted monitoring sites or time periods, various statistical techniques have been used to estimate reference conditions for the chemical composition of groundwater. In early work, reference conditions were determined as those concentrations lower than two standard deviations from the mean of all measured concentrations (Sinclair 1974; Nolan and Hitt 2003). As the mean was vulnerable to skewed data, subsequent refinement changed this to lower than two median absolute deviations from the median of all observed concentrations (Reimann et al. 2005). More recent work has focused on cumulative probability plots (Reimann et al. 2005; Panno et al. 2006; Koh et al. 2009). For example, using log-scale cumulative probability plots, changes in linear slopes are inferred to differentiate NO_3_–N concentrations that are indicative of reference conditions versus human impact, or to indicate different processes controlling groundwater quality (Coetsiers et al. 2009; Gemitzi 2012; Rahman et al. 2020). A related approach developed through the EU BRIDGE programme uses cumulative probability plots in concert with pre-selection methods to identify and exclude monitoring results that may show evidence of human impact, for example, based on parameters such as Cl concentration (Griffoen et al. 2008; Hinsby et al. 2008). Iterative outlier removal and gaussian mixture models can also be used to distinguish groundwater quality measurements that represent unimpacted versus impacted conditions (Nakić et al. 2007; Kim et al. 2015; Manu et al. 2022).

For streams, rivers and lakes, the reference conditions have also been inferred by isolating an anthropogenic factor such as the proportion of catchment area under intensive agriculture (Dodds and Oakes 2004; Abell et al. 2019). In surface waters, examples exist where variation is captured by classification systems (Snelder and Biggs 2002), resulting in several definitions of reference conditions. By grouping sites with similar characteristics and plotting contaminant concentrations at sites against, for example, upstream area in intensive agriculture, a regression can be fitted for each environmental class. Here the intercept represents reference conditions, because in this case it is predicting the chemical composition of freshwater in the absence of any upstream intensive agriculture (McDowell et al. 2013).

Each of the above-listed methods for evaluating reference conditions comes with advantages and disadvantages. Statistical methods for estimating reference conditions are fast, cheap, and can be relatively simple, but often suffer from higher uncertainty – especially if based on few measurements over large areas or complex geologies (Cruz and Andrade 2015). Moreover, these statistical methods do not necessarily provide unequivocal insight into the specific geochemical processes or anthropogenic drivers that are controlling the concentrations of NO_3_–N. In comparison, the use of isotopes and mass balance modelling is relatively expensive and time consuming but can produce robust definitions for reference conditions. Techniques that rely on environmental classifications and/or comparison of observed NO_3_–N concentrations to the levels of anthropogenic stressors, such as area of intensive agriculture, require information on the source areas (capture zones) of recharge, but this information is often lacking for groundwater monitoring sites. Of note for the context of the present investigation, relatively few previous studies have applied more than one method for evaluation of reference conditions for the chemical composition of groundwater, meaning that the strengths and weaknesses of the different techniques are difficult to compare and assess robustly.

The objective of this study is to estimate reference conditions and corresponding thresholds for NO_3_–N concentrations in New Zealand groundwater. This is undertaken using national-scale datasets and comparison of results from three methods. The first method is hierarchical cluster analysis (HCA), a multivariate statistical method applied by Daughney and Reeves (2005). The second method involves a comparison of NO_3_–N concentrations to groundwater residence times derived by fitting lumped parameter models to measurements of age tracers such as tritium, as demonstrated by Morgenstern and Daughney (2012). The final method employs a comparison of NO_3_–N concentrations to a metric of land use intensity in the area around each monitoring site, like the approach that has been applied to determine reference conditions for chemical indicators in New Zealand surface waters (McDowell et al. 2013). While all three methods have been applied previously in New Zealand, they have not employed data from the same sites and time periods, nor have they used consistent percentile-based thresholds that enable meaningful comparison of the inferred reference conditions.

## Methods

Calculations were performed with R version 4.2.2, using the specific functions and packages detailed hereafter.

### Groundwater quality data

**Figure 1.**
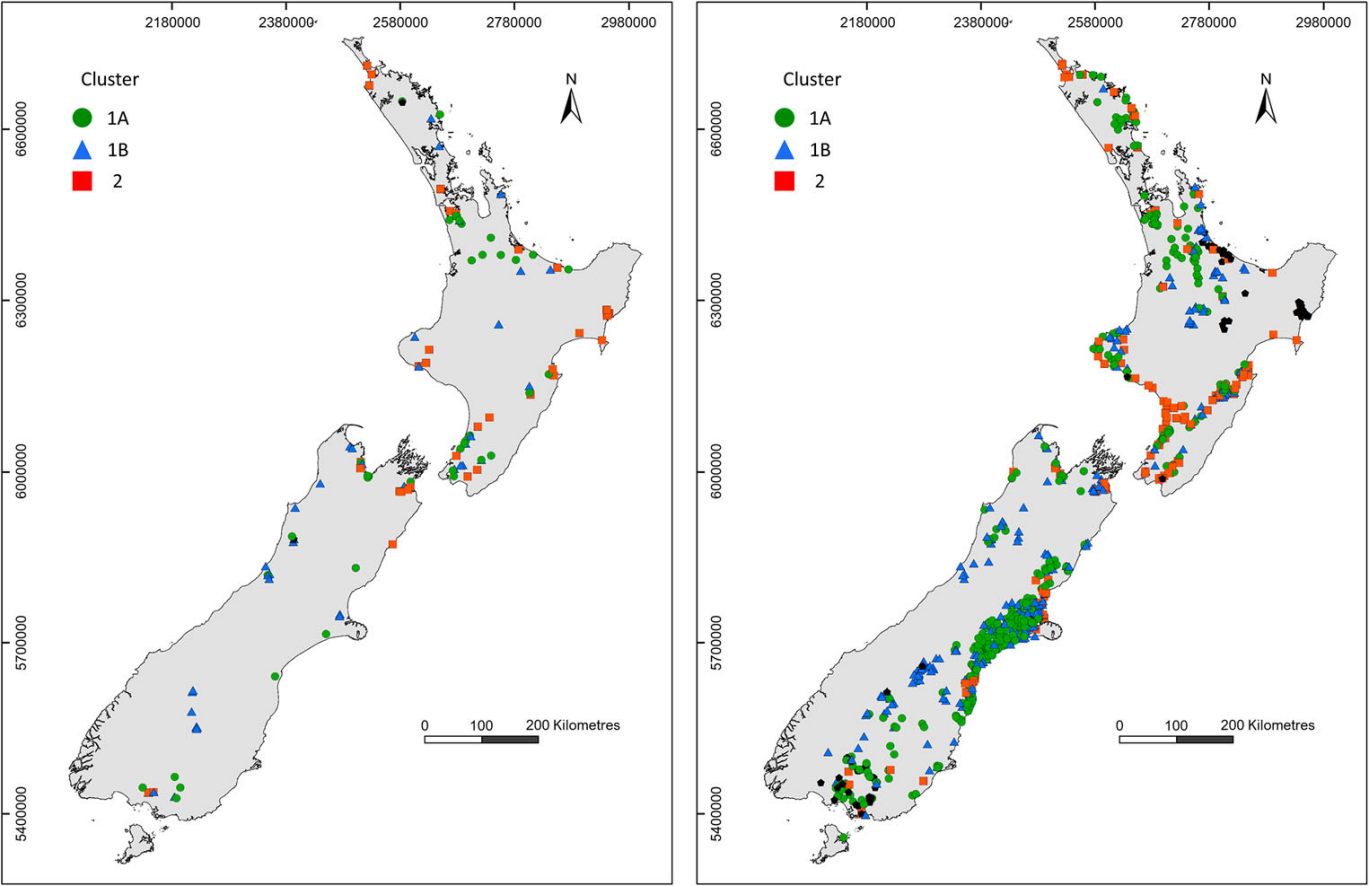
Locations of monitoring sites in the NGMP dataset (left panel) and LAWA dataset (right panel). Symbology corresponds to hydrochemical facies identified by HCA.

This study used two groundwater quality datasets (Figure 1). The first dataset was sourced from the New Zealand National Groundwater Monitoring programme (NGMP), a long-term research and monitoring programme that includes approximately 100 monitoring sites located across the country (Supplementary Material Table 1). NGMP sites are selected to encompass a range of land uses, aquifer confinement and lithology. Further details on the NGMP and its sites are provided by Daughney and Reeves (2005) and Daughney et al. (2012). The second dataset included measurements from 944 state-of-the-environment sites monitored by regional authorities and compiled by Land Air Water Aotearoa (LAWA). These state-of-the-environment monitoring sites are selected for a range of purposes according to the different monitoring objectives and network designs devised by the individual regional authorities.

Groundwater sampling and analytical methods are generally comparable between the NGMP dataset and the LAWA dataset (Daughney et al. 2012). Most sites are sampled quarterly, though some regional authority sites are monitored monthly or annually. Samples are collected according to a standard protocol (New Zealand Ministry for the Environment 2006). NGMP samples are analysed in the field for electrical conductivity, pH and temperature, and analysed in the laboratory for the concentrations of major ions (Na, K, Ca, Mg, HCO_3_, Cl, SO_4_) along with Br, F, Fe, Mn, SiO_2_ and selected forms of nitrogen and phosphorus (typically at least NO_3_–N, NH_4_–N and PO_4_–P). While many of these same parameters are also monitored by regional authorities (see Daughney and Randall 2009), only NO_3_–N, NH_4_–N, PO_4_–P, Cl and electrical conductivity are routinely made publicly available through the LAWA website (see Data Availability).

For the NGMP and LAWA datasets, this study used measurements made during two non-overlapping ten-year time periods: 1 January 2000 to 31 December 2009; and 1 January 2010 to 31 December 2019. Ten-year time periods were selected to provide sufficient data for analysis within each time window, particularly for estimation of upper and lower percentiles in the distributions of NO_3_–N concentration (Helsel et al. 2020). The start and end dates for these time windows are arbitrary but were selected such that the second period used in this study approximated the most recent intervals used for groundwater quality trend reporting by Statistics New Zealand (1999–2018; StatsNZ 2020) and LAWA (2012–2021; LAWA 2022).

For both time periods, the NGMP and the LAWA datasets contain censored analytical results (i.e. results reported as being below a detection limit), which is typical for water quality datasets (Helsel et al. 2020). The level of censoring varies by site, by parameter and over time. For example, censored results for Ca concentrations are very uncommon in the NGMP and LAWA datasets, whereas censored results account for a total of 20% and 11% of all NO_3_–N results in these two datasets, respectively, reflecting the very low concentrations that occur in some groundwaters (Langmuir 1997). Other parameters including F, Fe, Mn, NH_4_–N and PO_4_–P also commonly display censored results for a non-trivial proportion of sites and samples. For these parameters, the censoring threshold can change over time, e.g. due to improved analytical methods. To illustrate, across the NGMP and LAWA datasets there are more than ten different censoring thresholds for NO_3_–N, ranging from <0.001 to <1. The NGMP and LAWA datasets were therefore prepared for subsequent analysis using two steps appropriate for censored values as described below.

The first step in preparing the groundwater quality datasets for subsequent analysis was to calculate a median value for each parameter at each site, based on its available time-series data. This was undertaken separately for the two ten-year periods used in this study by applying the *ros* function of the *NADA* package to perform regression on order statistics. This approach uses log-transformation and Weibull plotting positions of uncensored values to replace the censored values with numeric estimates that are then used in the calculation of the site-specific median for a given parameter (Helsel et al. 2020). This approach is suitable for datasets with multiple censoring thresholds and censoring levels of up to 70%–80% (Helsel and Cohn 1988). In this study, the *ros* function was not applied to any site/parameter dataset that had a censoring level of greater than 70%, and instead the median was recorded as a censored value at the highest censoring threshold for the relevant parameter and site. As a hypothetical example, a site having measured concentrations of <0.005, <0.003 and <0.001 in three different samples would have its median recorded as <0.005. At the end of this data preparation step, the time-series datasets had been reduced to two *s* × *p* arrays (one array for the medians calculated for each ten-year period), where *s* and *p* were the number of sites and number of parameters in the dataset, respectively, and in which a portion of the results remained as censored values.

The second step in the data preparation was to replace the censored median values that remained after the first step. This was undertaken on a per-parameter basis whereby, across all sites, any remaining censored values were replaced with the highest censoring threshold for that parameter, and all uncensored values less than the same censoring threshold were similarly replaced (Helsel et al. 2020). For example, sites having median values of 0.22, 0.108, <0.03, 0.019 and <0.01 for a particular parameter would be replaced by 0.22, 0.108, 0.03, 0.03 and 0.03. The reason for this approach is that several of the methods used to estimate reference conditions cannot function with censored values, and it is not possible to determine which of these last three values is largest or smallest, hence they were treated as equivalent.

The impacts of these approaches for dealing with censored data are discussed below. Note that while the above method description refers to calculation of median values on a site/parameter basis, the same methods were also used to calculate the 80th percentiles used as input for estimation of reference conditions via regression against a measure of land-use impact.

### Estimating reference conditions

We estimated reference conditions for NO_3_–N in New Zealand groundwater using three separate approaches, each described in more detail in the following subsections: Hierarchical Cluster Analysis (HCA); groundwater age data; and a regression of NO_3_–N concentrations against a measure of land-use intensity. All approaches were applied independently to the NGMP dataset and the LAWA dataset, and independently for the two ten-year time periods (2000–2009 and 2010–2019).

To enable intercomparison of results, and to maintain consistency with other studies (e.g. Daughney and Reeves 2005; McDowell et al. 2013) and Government reporting (StatsNZ 2020), we describe reference conditions using seven percentile-based thresholds (5th, 10th, 20th, 50th, 80th, 90th, 95th) for each of the three above-listed methods. Unless otherwise noted, percentiles and their non-parametric 95% confidence intervals were determined using the *quantCI* function of the *quantileNPCI* package (version 1.6).

#### Hierarchical cluster analysis

HCA is a multivariate statistical method that has been employed to identify hydrochemical facies in groundwater systems (e.g. Güler et al. 2002). Each facies represents a group of groundwaters with similar chemical composition, for example, due to similar origin and/or pattern of hydrochemical evolution (Freeze and Cherry 1979). Identification of hydrochemical facies using HCA is typically based on the site-specific median values of many different water quality parameters, as shown through the early work of Back (1961, 1966), Morgan and Winner (1962) and Seaber (1962). In this way, the set of hydrochemical facies identified in a large water quality dataset provides a convenient summary of a large amount of multivariate time-series data, while also providing insights into key factors that may drive hydrochemical evolution, such as water-rock interaction, aquifer flow pathways, groundwater-surface water interaction and extent of human impacts (e.g. Farnham et al. 2002; Cloutier et al. 2008; Guggenmos et al. 2011; Raiber et al. 2012; Nejatijahromi et al. 2019). Note that HCA does not provide direct information on the processes or drivers that differentiate the hydrochemical facies, so these must be inferred through interpretation and/or other sources of information. Nonetheless, previous work in New Zealand has demonstrated that hydrochemical facies identified through HCA can be used to infer reference conditions for groundwater quality (Daughney and Reeves 2005; Daughney et al. 2012).

In this study we evaluate hydrochemical facies in New Zealand groundwater using the NGMP dataset. HCA was conducted using the *hclust* command (contained in the R base *stats* package), based on z-scored, log-transformed site-specific median concentrations of fifteen parameters (Br, Ca, Cl, F, Fe, HCO_3_, K, Mg, Mn, Na, NH_4_–N, NO_3_–N, PO_4_–P, SiO_2_, SO_4_). Clustering was based on Ward’s method, with the square of the Euclidean distance used as the separation measure (Güler et al. 2002). These methods are identical to those of Daughney and Reeves (2005), except that in the earlier study the site-specific medians were based on samples collected over different time periods, starting at whatever date each site joined the NGMP (between 1990 and 1995) and ending in 2003, whereas the present study uses the two 10-year time periods (2000–2009 and 2010–2019) as noted above.

Identical HCA methods could not be applied to the LAWA dataset because it only includes analyses of NO_3_–N, NH_4_–N, PO_4_–P, Cl and electrical conductivity. However, the sites comprising the LAWA dataset are known to display the same hydrochemical facies as the NGMP dataset (Daughney et al. 2012). Thus, we used the NGMP dataset to train a linear discriminant analysis (LDA) model using the *lda* function in the package *MASS* (version 7.3–58.1). The LDA model was trained to predict each NGMP site’s assignment to a hydrochemical facies, but based only on its z-scored, log-transformed median concentrations of the five water quality variables that are also available in the LAWA dataset. This approach is similar to previous applications of the NGMP dataset for training of machine-learning applications (Moreau and Daughney 2021). Next, we validated the LDA model by testing its ability to accurately reproduce hydrochemical facies assignments previously reported by Daughney and Randall (2009) for a national dataset of over 1000 groundwater sites, many of which are included in the LAWA dataset. Finally, we applied the validated LDA model to assign each site in the LAWA dataset to one of the hydrochemical facies recognised in the NGMP dataset.

#### Relationship to groundwater age

Groundwater age describes the residence time of a parcel of groundwater within an aquifer system, i.e. the time elapsed since recharge. Due to convergence of flow paths of different length and recharge source, mainly at the sampled discharge points such as wells and springs, any groundwater sample contains a mixture of different ages (Maloszewski and Zuber 1996). The groundwater age distribution within a particular sample can be described by measures of its central tendency (e.g. mean age) and distributional shape (e.g. age mixing model), which in turn can be estimated by fitting a lumped parameter model (LPM) to measured concentrations of age tracers in the groundwater sample (Zuber et al. 2005; Daughney et al. 2010; Morgenstern and Daughney 2012).

Groundwater age distributions for the NGMP sites were first reported by Daughney et al. (2010). The age distributions were evaluated by fitting the exponential-piston flow LPM (Zuber et al. 2005) to site-specific concentrations of the age tracers tritium, sulphur hexafluoride (SF_6_) and chlorofluorocarbons (CFCs) measured in the period 2005–2010. Interpreted mean groundwater age at the NGMP sites ranged from less than 1 year to more than 100 years, with the 25th, 50th and 75th percentiles in mean groundwater age across the NGMP network being approximately 10, 40 and 100 years, respectively.

Morgenstern and Daughney (2012) compared the mean groundwater age at the NGMP sites to the site-specific median concentrations of a range of groundwater quality parameters as reported by Daughney and Randall (2009). Concentrations of parameters such as Na, HCO_3_, SiO_2_, F, Fe, Mn and PO_4_–P were generally observed to increase with groundwater age, reflecting likely origin from natural water-rock interaction. Concentrations of other parameters such as NO_3_–N, Cl and SO_4_ were generally observed to be higher in younger groundwaters, suggesting that they may be sourced from human activities and/or subject to reactions that tend to cause their concentrations to decrease over time (e.g. denitrification). Morgenstern and Daughney (2012) used a visual fitting approach to infer reference conditions based on the observed relationships between groundwater quality and mean age.

In the present study we re-plot the relationships between groundwater age and NO_3_–N concentration using updated data from the NGMP network. Site-specific median NO_3_–N concentrations were determined for the two periods (2000–2009, 2010–2019) to allow comparison with results from other methods used in this study. The groundwater age distributions reported by Morgenstern and Daughney (2012) were reinterpreted using TracerLPM (version 1) (Jurgens et al. 2012) by re-fitting LPMs to account for time-series age tracer measurements made since 2010 (Supplementary Material Table 1). Improved age interpretations were developed for most sites, made possible from availability of additional complementary tracers (SF_6_, Halon-1301) and longer time series of tracer data, and the fact that the so-called bomb-tritium from the atmospheric thermonuclear weapons testing in the early 1960s has further decayed, removing any ambiguity in data interpretation over the last 10 years in New Zealand. Of note, the richness of the longer time series and additional tracer data now available reveals the existence of binary age mixing at some NGMP sites (cf. Morgenstern et al. 2015), for example due to wells tapping into two aquifers and subsequent mixing of water with a younger and an older age distribution, necessitating the application of more complex LPMs than used previously, and providing greater constraint on the site-specific mean groundwater age.

As an improvement over the visual fitting approach used by Morgenstern and Daughney (2012), in this study we used the constraint line method to interpret reference conditions and coinciding thresholds from the plot of site-specific mean groundwater age versus median NO_3_–N concentration. The constraint line was determined by dividing the plot into bins along the x-axis, determining the 99th quantile for each bin, then fitting a regression line to these points (Hao and Wu 2017). An optimal bin size of 25 years was selected to provide a balance between maximising the number of bins while retaining a sufficient number of data points in each bin to allow estimation of the 99th quantile. For comparison we used a second approach, which ignored the constraint line and instead applied quantile regression at the 80th percentile for sites assigned to the same hydrochemical facies.

Due to the lack of an available compilation of groundwater age distributions, the LAWA dataset could not be used to validate the results obtained from the NGMP dataset with this method.

#### Regression against a measure of land-use impact

Regression of observed concentrations against a measure of land-use impact has been applied to estimate the reference conditions for chemical indicators in New Zealand rivers and streams (McDowell et al. 2013). In this approach, the 80th percentile for NO_3_–N concentration was calculated from time series data for each of >1000 river water monitoring sites across New Zealand. A national river environment classification (REC) was applied to group the sites into categories according to the environmental conditions that are strong determinants of water quality (Snelder and Biggs 2002). For each of the main REC classes, the site-specific values for the 80th percentile NO_3_–N concentration were plotted against the percentage of intensive agricultural land use in the catchment upstream of each site. Here, intensive agricultural land use represents an indicator of human activity. Fewer than 10% of the monitoring sites were situated in pristine unmodified catchments so, for each of the main REC classes, the intercept of a fitted regression line was used to estimate the 80th percentile NO_3_–N concentration expected for sites with no intensive agriculture upstream. These values have been subsequently adopted as Default Guideline Values (under reference conditions) for New Zealand rivers in the Australian and New Zealand Guidelines for Fresh and Marine Water Quality (ANZG 2018).

In the present study we applied an analogous approach using the NGMP dataset to estimate reference conditions for the concentration of NO_3_–N in New Zealand groundwater. To estimate the degree of land-use impact affecting each site, we used a national map of estimated NO_3_–N leached from livestock in the 2017 year (StatsNZ 2019), which accounts for animal types, stocking density and physiographic factors such as soil type and climate, but does not quantify uncertainty (Ausseil and Manderson 2018). Note that the mapped leaching rates do not account for fertiliser application or non-agricultural sources of NO_3_–N, but livestock sources are dominant in New Zealand (Parfitt et al. 2012). To date, the land areas contributing recharge (i.e. capture zones) have been rigorously mapped for only a small number of NGMP sites (e.g. Toews and Donath 2015), so in the absence of this information we determined the average NO_3_–N leaching rate (kg N per ha) within a defined ‘circular area surrogate’ using a 0.5, 1.0, 2.0 or 5.0 km radius around each site (Johnson and Belitz 2009; Wheeler et al. 2015). For this circular area around each site, we determined the 80th percentile in the observed NO_3_–N concentration in groundwater for the period 2010–2019, to correspond with the 2017 year used for the NO_3_–N leaching map.

The approach described above was applied firstly by considering all the NGMP sites together in a single group. In the study by McDowell et al. (2013), a spline was included in the regression to assess the relationship between NO_3_–N concentration and land-use intensity because most sites had high land-use impacts, meaning that the few sites with low land-use impact may have had insufficient leverage to allow accurate estimation of the intercept by linear regression. The NGMP dataset has a more even distribution of sites with high, medium and low land-use impacts, so linear regression slope was seen as fit for evaluating the relationship between groundwater NO_3_–N concentration and land-use intensity, whereby the regression intercept and its confidence intervals were determined to estimate the 80th percentile for NO_3_–N concentration in groundwater in the absence of any NO_3_–N leached from livestock.

We performed a second evaluation in which the NGMP sites were segregated into hydrochemical facies derived from HCA, and each site’s weighting in the regression was scaled according to its fraction of groundwater aged 5 years or less. This age-weighting approach was followed to maximise the regression influence for those sites with residence time distributions most closely corresponding to the 2017 year used for the NO_3_–N leaching map.

The methods applied to the NGMP dataset were then re-applied to the LAWA dataset, except for the age-weighting approach, which was prevented by the lack of a compilation of age data from the LAWA sites.

#### Integrating results from different methods

We integrated the results from selected methods, time periods and datasets to estimate reference conditions for NO_3_–N in New Zealand groundwater. The first step was to evaluate the strengths and weaknesses of the different methods, time periods and datasets, and select a subset of results with sufficient robustness for inclusion in the estimation of reference conditions. The second step was to use a weight of evidence approach (Linkov et al. 2009; Hope and Clarkson 2014; USEPA 2016) to calculate selected percentiles (5th, 10th, 20th, 50th, 80th, 90th, 95th) in the NO_3_–N concentration under reference conditions. By this method, a weighted average was determined for each percentile based on the results obtained from the different methods, time periods and datasets, whereby the contribution of a particular result was weighted by the inverse of its confidence interval to give higher influence to those results with lower uncertainty.

## Results and discussion

### Hierarchical cluster analysis

**Figure 2.**
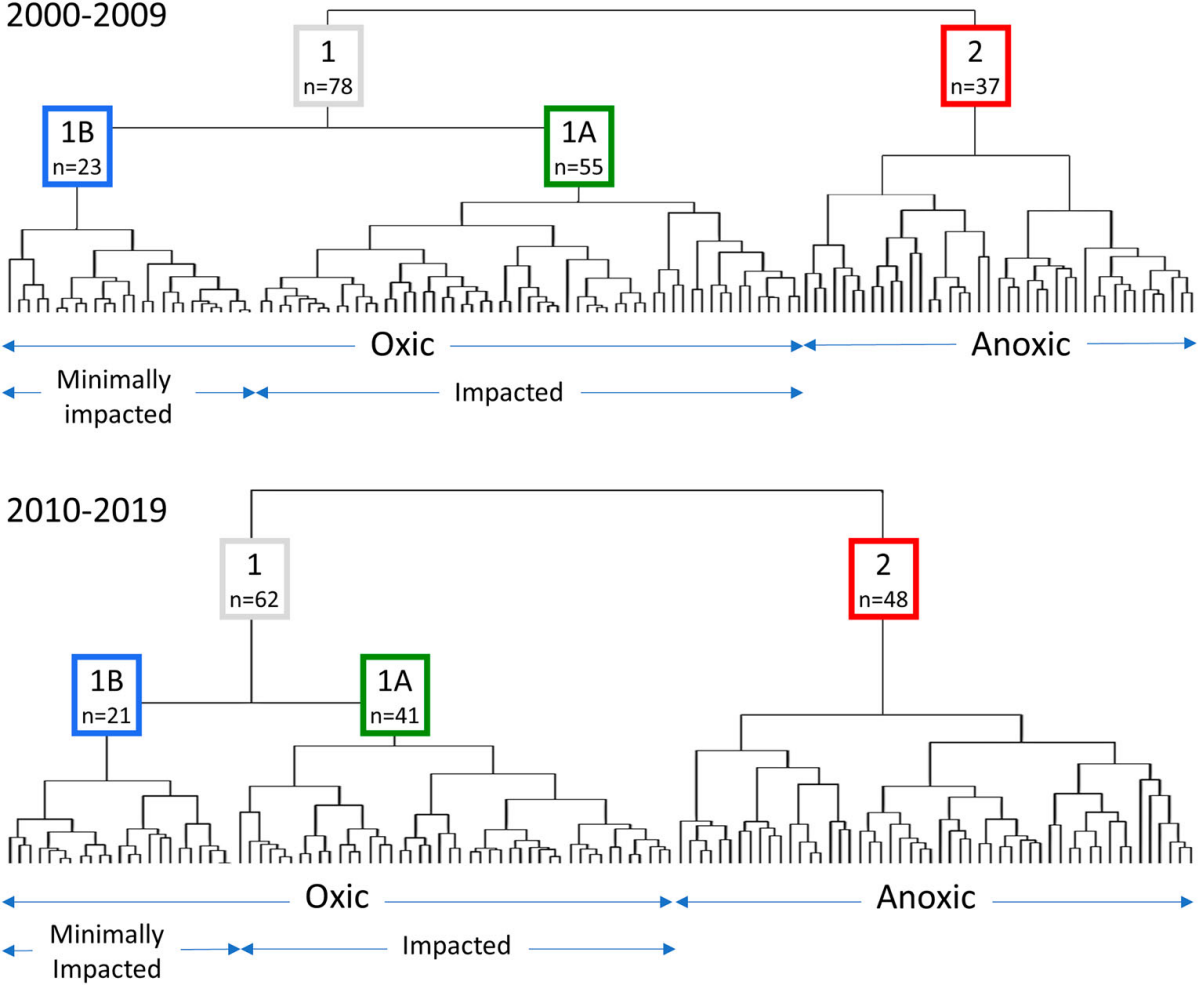
Dendrogram for the NGMP dataset based on HCA conducted with site-specific median concentrations of Br, Ca, Cl, F, Fe, HCO_3_, K, Mg, Mn, Na, NH_4_–N, NO_3_–N, PO_4_–P, SiO_2_ and SO_4_ for samples collected in the period 2000–2009 (top panel) or 2010–2019 (bottom panel). Terminus of each vertical line along x-axis represents one NGMP site. Labels correspond to hydrochemical facies described in the text and number of sites (n) assigned to them.

HCA conducted with the NGMP dataset identified three hydrochemical facies of relevance to the present study (Figure 2). At the highest HCA separation threshold, the NGMP sites were partitioned into Cluster 1 and Cluster 2, representing oxic and anoxic groundwaters, respectively. Groundwater sites assigned to Cluster 1 were typified by concentrations of NO_3_–N that were above the analytical detection-limit and concentrations of NH_4_–N, Fe and Mn that were near or below their analytical detection limits, whereas sites assigned to Cluster 2 displayed the opposite pattern. The shifts in the concentrations of these redox-sensitive substances reflect their expected behaviour in response to aquifer microbial processes (Chapelle 2000) and natural water-rock interaction (Langmuir 1997). At a lower HCA separation threshold, the oxic groundwaters were subdivided into Cluster 1A and Cluster 1B, interpreted to represent groundwaters that were impacted or minimally impacted by human activity, respectively. We infer that the degree of human impact is a primary driver that differentiates Cluster 1A from Cluster 1B, as evidenced by the former cluster’s typically higher concentrations of NO_3_–N, sometimes also accompanied by elevated concentrations of other substances such as K, Na and/or Cl. Further lowering the HCA separation threshold allows additional subclusters to be identified, which within Cluster 1B are inferred to be driven by aquifer lithology, i.e. clastic or carbonate versus volcanic or volcaniclastic (Daughney and Reeves 2005); however, these subclusters are not strongly differentiated by their NO_3_–N concentrations so are not discussed in this paper.

These three hydrochemical facies represented by Clusters 1A, 1B and 2 in the NGMP dataset are very similar to those previously identified using the same HCA methods by Daughney and Reeves (2005) and later by Daughney et al. (2012). Repeated identification of these same main hydrochemical facies over different time periods likely occurs because groundwater chemistry is relatively static or otherwise changing slowly at most NGMP sites (Daughney and Reeves 2006; Moreau and Daughney 2021). For the sites where groundwater quality has changed more significantly over time, it tends to shift from one to another of the three hydrochemical facies described in the preceding paragraph, rather than towards some previously unrecognised hydrochemical facies. This suggests that water-rock interaction, aquifer redox processes and human impacts are overarching and enduring controls on groundwater quality in New Zealand.

Using the three main hydrochemical facies identified by HCA with the NGMP data collected up to the end of 2003, Daughney and Reeves (2005) interpreted that reference conditions for NO_3_–N in oxic, minimally impacted groundwater in New Zealand could be defined based on the sites assigned to Cluster 1B. Noting that reference conditions must be defined as a range to reflect natural variation, Daughney and Reeves (2005) proposed that NO_3_–N concentrations above the 75th percentile or 95th percentile observed for Cluster 1B were ‘probably’ and ‘almost certainly’ indicative of human impact, respectively, for which the corresponding NO_3_–N thresholds were 1.6 and 3.5 mg/l (the 80th percentile was 1.81 mg/l, though this particular threshold was not originally reported). Data from NGMP sites that were assigned to Cluster 1A were inferred to show a significant degree of human impact and so were not used for the estimation of reference conditions for oxic groundwaters. Data from NGMP sites assigned to Cluster 2 were typified by reduced groundwater with low or below-detection concentrations of NO_3_–N so were likewise excluded from the estimation of reference conditions for oxic groundwater. However, noting that reduced groundwaters can occur naturally, thresholds were reported separately for NO_3_–N in anoxic groundwater (Cluster 2), namely as 0.02 and 0.05 mg/l for the 75th and 95th percentiles, respectively.

Following the same approach using the NGMP dataset in the present study and the 80th percentile as an example, we calculated the corresponding NO_3_–N concentration in oxic minimally impacted groundwater (Cluster 1B) to be 1.70 ± 0.23 mg/l if based on samples collected in the decade 2000–2009, or 1.44 ± 0.24 mg/l if based on samples collected in the decade 2010–2019 (see Table 1 for other selected percentiles). For comparison, the 80th and 90th percentiles for NO_3_–N concentration in anoxic groundwater (Cluster 2) were found to be 0.04 ± 0.01 and 0.18 ± 0.02 mg/l (2000–2009 period), or 0.03 ± 0.01 and 0.13 ± 0.02 mg/l (2010–2019 period), respectively (95th percentiles could not be estimated robustly). That the selected percentiles in NO_3_–N concentration for each hydrochemical facies changes relatively little between the two ten-year periods used in the present study, or in comparison to the earlier study of Daughney and Reeves (2005), is to be expected if these facies do in fact represent minimally impacted groundwaters, which would therefore not be affected by changing loadings of NO_3_–N arising from temporal changes in use of land or water in the area around the monitoring sites.

**Table 1.** Selected percentiles ± 95% confidence intervals for NO_3_–N concentration (mg/l) in oxic, minimally impacted groundwater as determined in this study using data from two time periods for the NGMP and LAWA datasets.

Method	Sub-Method	Percentile	NGMP Dataset		LAWA Dataset	
			2000–2009	2010–2019	2000–2009	2010–2019
Hierarchical cluster analysis		5th	0.14 ± 0.09	0.14 ± 0.09	0.07 ± 0.07	0.06 ± 0.07
		10th	0.16 ± 0.10	0.17 ± 0.10	0.13 ± 0.09	0.11 ± 0.08
		20th	0.28 ± 0.13	0.29 ± 0.11	0.23 ± 0.11	0.20 ± 0.12
		50th	0.74 ± 0.18	0.76 ± 0.20	0.66 ± 0.19	0.65 ± 0.21
		80th	1.70 ± 0.23	1.44 ± 0.32	1.65 ± 0.28	1.68 ± 0.31
		90th	2.10 ± 0.30	1.88 ± 0.25	2.68 ± 0.31	2.61 ± 0.36
		95th	2.50 ± 0.28	2.37 ± 0.24	3.36 ± 0.51	3.50 ± 0.43
Comparison to groundwater age	Constraint line method based on sites assigned to Clusters 1A and 1B (oxic groundwaters)	5th	0.14 ± 0.07	0.14 ± 0.08	N/A	
		10th	0.16 ± 0.08	0.17 ± 0.10		
		20th	0.28 ± 0.11	0.29 ± 0.15		
		50th	0.74 ± 0.21	0.76 ± 0.19		
		80th	1.79 ± 0.32	1.79 ± 0.35		
		90th	1.92 ± 0.36	1.99 ± 0.40		
		95th	2.10 ± 0.44	2.15 ± 0.44		
	Quantile regression based on sites assigned to Cluster 1B (oxic minimally impacted groundwaters)	5th	0.13 ± 0.05	0.14 ± 0.05		
		10th	0.16 ± 0.04	0.17 ± 0.12		
		20th	0.28 ± 0.13	0.30 ± 0.13		
		50th	0.78 ± 0.27	0.70 ± 0.32		
		80th	1.61 ± 0.60	1.61 ± 0.53		
		90th	2.16 ± 0.47	2.26 ± 0.61		
		95th	2.29 ± 0.35	2.31 ± 0.49		
Regression against a measure of land-use impact	Linear regression based on all sites	5th	N/A	0.64 ± 0.72	N/A	1.40 ± 0.32
		10th		0.70 ± 0.69		1.49 ± 0.33
		20th		0.77 ± 0.67		1.61 ± 0.34
		50th		1.15 ± 0.67		1.89 ± 0.38
		80th		1.42 ± 0.53		2.23 ± 0.43
		90th		1.54 ± 0.66		2.45 ± 0.45
		95th		1.69 ± 0.97		2.64 ± 0.48
	Linear regression based on sites assigned to Cluster 1A (oxic minimally impacted groundwaters)	5th	N/A	N/A	N/A	4.48 ± 0.60
		10th		0.81 ± 0.41		4.81 ± 0.62
		20th		0.96 ± 0.34		5.07 ± 0.64
		50th		1.20 ± 1.01		5.88 ± 0.70
		80th		1.87 ± 1.63		6.73 ± 0.77
		90th		2.07 ± 1.59		7.17 ± 0.79
		95th		2.16 ± 1.69		7.60 ± 0.84

Grey shaded cells contain values used to calculate the weighted averages for reference conditions compiled in Table 2.

The same HCA methods cannot be applied to the LAWA dataset because it only includes analyses of NO_3_–N, NH_4_–N, PO_4_–P, Cl, and electrical conductivity. Therefore, we used the LDA model to predict each site’s hydrochemical facies based only on these available parameters. The LDA model was initially calibrated using the NGMP dataset and was able to correctly apportion 90% of the NGMP sites to their originally assigned clusters (1A, 1B, 2). Next, we confirmed that the LDA model was able to achieve >80% accuracy in predicting the hydrochemical facies assignments previously reported by Daughney and Randall (2009) for a national dataset of over 1000 regional council groundwater monitoring sites. Using the validated LDA model applied to the LAWA sites, we calculated that the 80th percentile for NO_3_–N concentration in oxic, minimally impacted groundwater (Cluster 1B, *n* = 278) is 1.65 ± 0.28 mg/l if based on samples collected in the decade 2000–2009, and 1.68 ± 0.31 mg/l if based on samples collected in the decade 2010–2019 (Table 1). These results are in good agreement with the 80th percentile values determined from the NGMP dataset for these same time periods. For anoxic groundwater (Cluster 2) the 80th percentile NO_3_–N concentration was found to be 0.03 ± 0.01 mg/l for the period 2000–2009 and 0.04 ± 0.01 mg/l for the period 2010–2019, whereas the 90th percentiles were 0.05 ± 0.02 mg/l for both time periods (results not tabulated). We caution that requirement to use an LDA model to predict cluster assignments means that the percentile values derived from the LAWA dataset should not be used beyond providing general validation of the results from the NGMP dataset.

We note certain advantages and limitations of HCA for estimating reference conditions for groundwater quality. One advantage is that the identification of minimally impacted groundwater is based on several parameters (e.g. Na, K, Cl) in addition to NO_3_–N. This means that groundwater that is only marginally degraded based on its NO_3_–N concentration can still be identified as impacted based on other indicators, thereby allowing for added sensitivity that a univariate method may miss. A key limitation of the HCA method is that it does not provide any definitive information about the actual processes that differentiate the clusters; instead, these drivers can only be inferred, which may be difficult if there are several interacting processes involved. For example, the distinction between Clusters 1A and 1B may be due not only to the degree of human impact, but also to age of the groundwater or the relative proportions of groundwater recharge that a site receives from rainfall passage through the soil zone compared to seepage from rivers (Daughney and Reeves 2005). Some of these limitations are addressed with the method applied in the following section.

### Relationship to groundwater age

Groundwater age distribution parameters for the NGMP sites are provided in Supplementary Material Table 1, based on fitting of LPMs to site-specific time-series measurements of several age tracers, mainly tritium, SF_6_ and chlorofluorocarbons. For most sites, the mean groundwater age inferred in this study is comparable to that originally reported by Daughney et al. (2010). However, significant differences in the LPM-derived mean groundwater age do occur for some sites, typically because the additional age tracer data collected since 2010 now allow for constraint of more complex age distributions, e.g. binary mixing models due to convergence of old and young water flow paths at the sampled discharge sites. There is also a small minority of NGMP sites at which the groundwater age distribution is interpreted to have shifted over time, for example due to changes in rates of recharge or abstraction that could affect flows of groundwater through some parts of some aquifer systems.

**Figure 3.**
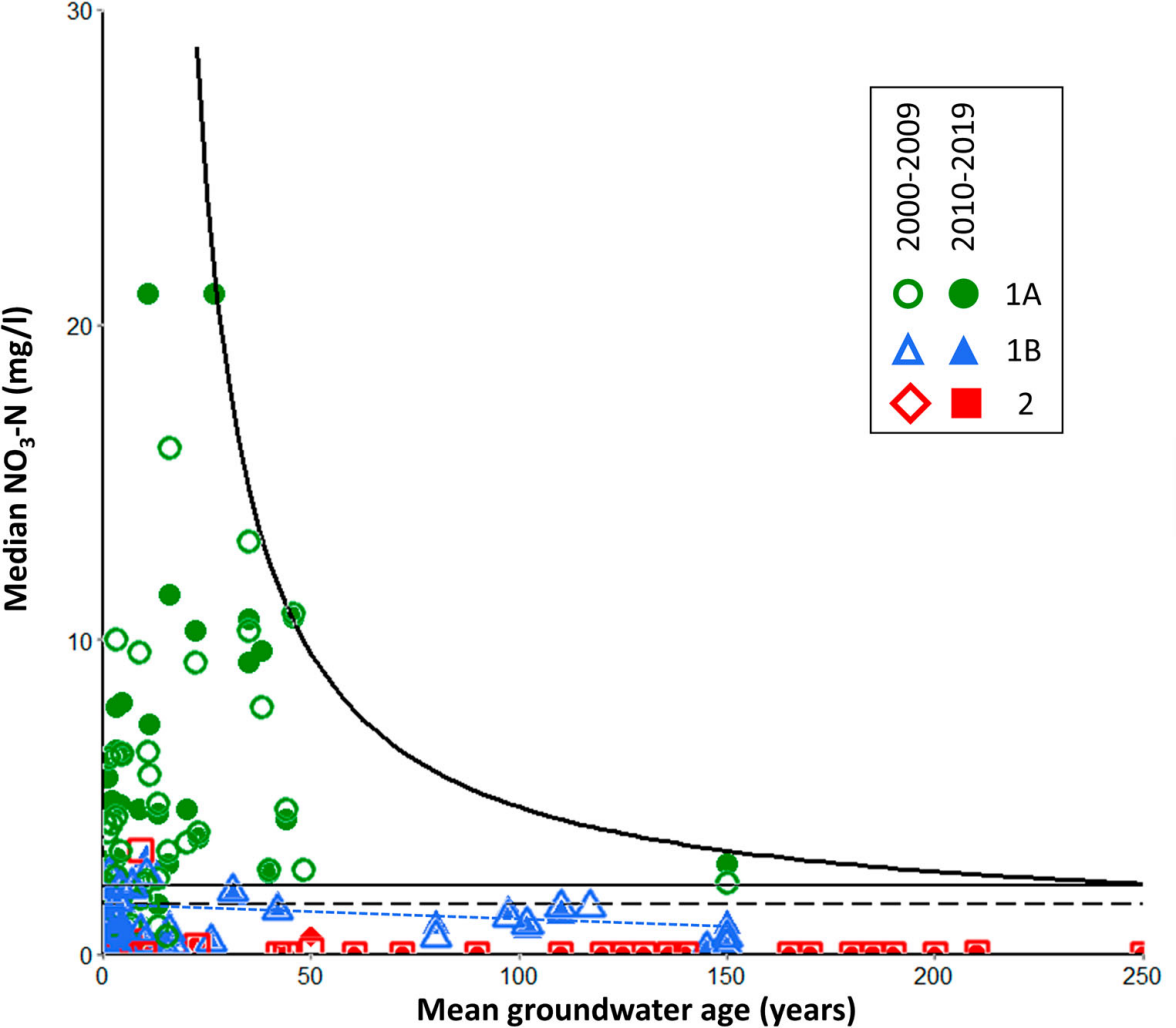
Median NO_3_–N concentration for the periods 2000–2009 (open symbols) and 2010–2019 (closed symbols) versus inferred mean groundwater age for sites in the NGMP dataset. Symbol colours represent hydrochemical facies. Black lines are derived from the constraint line method: solid hyperbola is the fitted constraint line; solid and dashed horizontal lines indicate the asymptote of the constraint line and the 80th percentile for oxic groundwater, respectively. Blue dotted line is derived from the quantile regression method, indicating the 80th quantile for all Cluster 1B groundwaters.

Figure 3 displays the relationships between mean groundwater age and the median NO_3_–N concentrations for the NGMP sites. The highest concentrations of NO_3_–N are found in sites with young, oxic groundwaters that show evidence of human impact based on HCA (Cluster 1A). Groundwaters that are oxic but minimally impacted (Cluster 1B) typically have lower but detectable concentrations of NO_3_–N, even for sites with mean groundwater age >100 years. Regardless of mean age, anoxic groundwaters tend to have very low concentrations of NO_3_–N. These general relationships between mean groundwater age and NO_3_–N concentration have been previously reported for the NGMP sites (Morgenstern and Daughney 2012).

Estimation of reference conditions for NO_3_–N via its relationship to mean groundwater age was first reported by Morgenstern and Daughney (2012) using a visual graph fitting approach. A threshold of >0.2 mg/l was identified for the NGMP dataset as separating reference conditions for unimpacted oxic groundwater versus groundwater affected by ‘low intensity land-use’. We note that this threshold was defined based on only three NGMP sites. A second threshold of 2.5 mg/l NO_3_–N was identified to differentiate oxic groundwaters impacted by ‘low-intensity’ versus ‘high intensity’ land-use. No thresholds were reported for NO_3_–N in anoxic groundwater.

In the present study, we estimated reference conditions for NO_3_–N using a constraint line approach (Hao and Wu 2017). The best-fitting constraint line is a hyperbolic equation with a maximum NO_3_–N concentration that approaches an asymptote of 2.2 mg/l as mean groundwater age approaches 250 years (Figure 3), which is towards the upper end of values reported for the NGMP dataset and the effective limit of interpretability of LPMs based on the age tracers used in this study (Morgenstern and Taylor 2009; Morgenstern and Daughney 2012). Once the constraint line had been fitted, we determined selected percentiles for NO_3_–N concentration in oxic minimally impacted groundwater using all oxic groundwaters (Clusters 1A and 1B) that plot below the horizontal asymptote of the constraint line. Again, using the 80th percentile as an example, the threshold for oxic groundwater is determined to be 1.79 ± 0.32 mg/l for the 2000–2009 period, and 1.79 ± 0.35 mg/l for the 2010–2019 period (Table 1). We note that there is a possible bias in this approach because the calculation includes some sites that are inferred to show evidence of human impact based on HCA (i.e. sites assigned to Cluster 1A). This constraint line approach cannot be used to estimate percentiles in the NO_3_–N concentrations for anoxic groundwater.

For comparison we used the NGMP dataset for a second approach, which ignores the constraint line and instead applies quantile regression using only those sites assigned to Cluster 1B, i.e. oxic and minimally impacted according to HCA. The slope of the quantile regression line is not significantly different from zero (*p* > 0.05) for either period, so the intercept is taken as the threshold concentration of NO_3_–N in oxic minimally impacted groundwater. Again, using the 80th quantile as an example, the regression line’s intercept was determined to be 1.61 ± 0.60 for data from the 2000–2009 period, or 1.61 ± 0.53 for data from the 2010–2019 period (Table 1). This approach can also be applied to anoxic groundwater (Cluster 2), which yielded estimates of the 80th and 90th percentiles in NO_3_–N concentration of 0.05 ± 0.02 and 0.30 ± 0.07 (2000–2009 period), or 0.05 ± 0.01 and 0.35 ± 0.15 (2010–2019 period), respectively (results not tabulated); 95th percentiles could not be estimated robustly.

Due to the lack of an available compilation of their groundwater age distributions, the LAWA dataset cannot presently be used to validate the results from the NGMP dataset. However, some regional and catchment-scale investigations have been undertaken and reveal generally similar relationships between groundwater quality and groundwater age as observed in the NGMP dataset (e.g. Morgenstern et al. 2015, 2018, 2019). For example, concentrations of parameters such as Na, HCO_3_, SiO_2_, Fe, Mn and PO_4_–P have generally been observed to increase with groundwater age, reflecting likely origin from natural water-rock interaction, whereas concentrations of parameters such as NO_3_–N, Cl and SO_4_ have generally been observed to be higher in younger groundwaters, suggesting that they may be sourced from human activities and/or subject to reactions that tend to cause their concentrations to decrease over time (e.g. denitrification, sulphate reduction).

We note certain advantages and limitations of estimating reference conditions for groundwater quality based on its relationship to groundwater age. One advantage is that groundwater age can be helpfully compared to the history of land use in the vicinity of the monitoring site, which provides constraint on the level of human impact and therefore the NO_3_–N concentration that might be expected. We point out that the LPMs applied in this study follow the assumption that the groundwater age distribution at each site is constant over the long term, despite seasonal fluctuations that may exist at some sites (e.g. Toews et al. 2016). Constancy of groundwater age distribution is a reasonable assumption for New Zealand groundwater systems, which experience temperate climates and are not generally highly exploited, but we acknowledge that this assumption may be violated if this method is applied elsewhere. A disadvantage of the groundwater age approach is that, as a univariate method, the relationships to groundwater quality are explored only one parameter at a time. However, as shown in this study, this limitation can be overcome by categorising the monitoring sites using a multivariate hydrochemical method such as HCA. Another disadvantage of the groundwater age method is that, like HCA, it does not provide any definitive information about the actual land-use intensity that occurred in the vicinity of a well at the time of recharge. This limitation is addressed with the method applied in the following section.

### Regression against a measure of land-use impact

**Figure 4.**
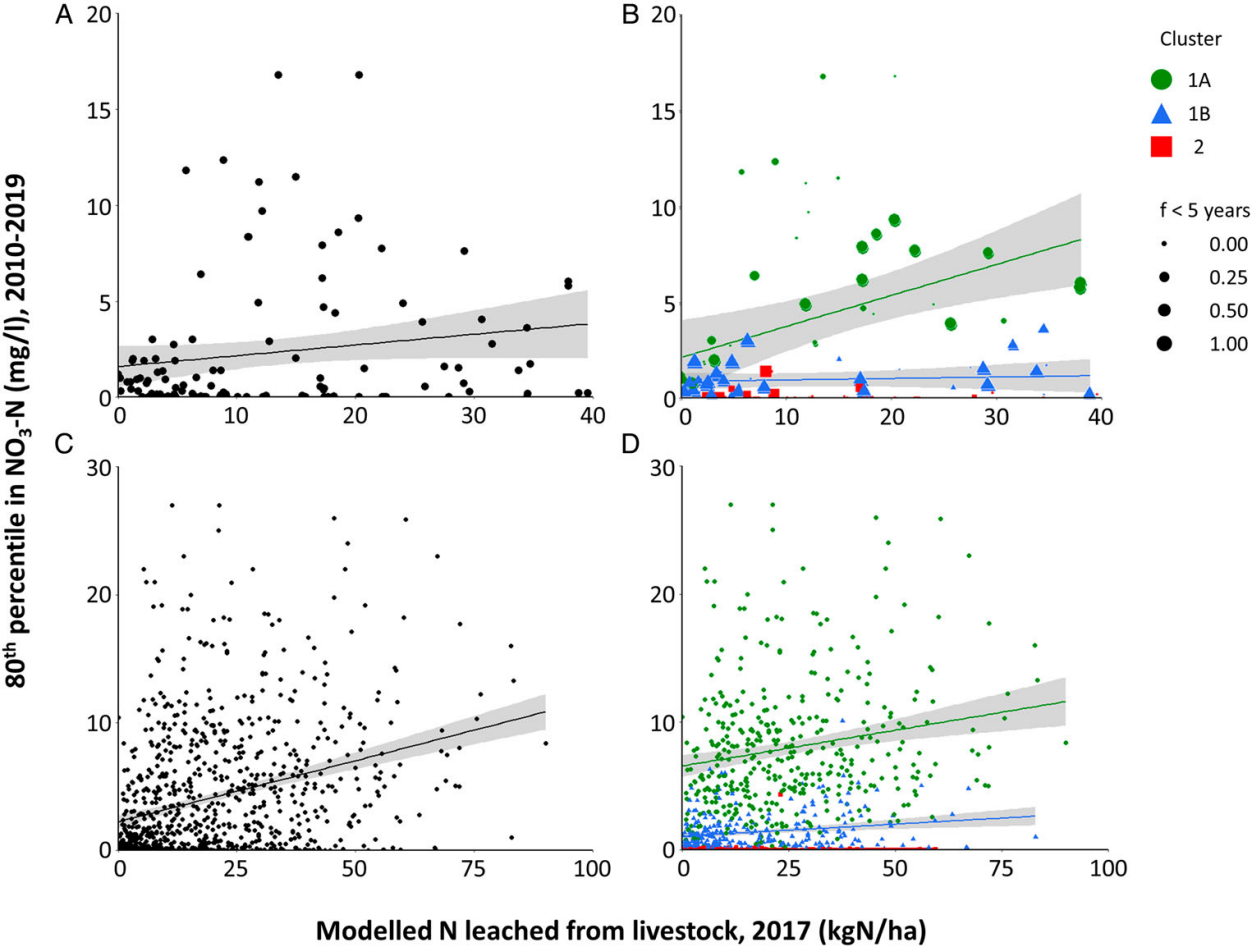
80th percentile in NO_3_–N concentration for the period 2010–2019 versus average modelled NO_3_–N leached from livestock in 2017 within a 2 km radius of each site (note differences in axis scales), with corresponding linear regression lines and 95% confidence limits (shaded areas): **A**, All NGMP sites plotted together as a single group. **B**, NGMP sites segregated by hydrochemical cluster with individual regression lines; symbol size is scaled to the fraction of groundwater that has age less than 5 years. **C**, All LAWA sites plotted together as a single group. **D**, LAWA sites segregated by hydrochemical clusters predicted by LDA.

Figure 4 displays plots of the 80th percentile in the measured NO_3_–N concentration in groundwater at each site compared to the average NO_3_–N leaching rate in a circular area around that site (similar plots were generated for other percentiles, not shown). The results were not significantly different for circular area radii from 0.5 to 5.0 km (*p* > 0.1), so all following results are based on a radius of 2.0 km. We are unable to accurately quantify the uncertainties in the average NO_3_–N leaching rates depicted in Figure 4 because such uncertainties were not reported by Ausseil and Manderson (2018). These data were generated using a Nutrient Budget model (Overseer), whose uncertainty based on changing climate and soil data is estimated to be 27 ± 9% (Tavernet and Harper 2022), and we acknowledge that modelled NO_3_–N leached from land can be highly variable within and between farms according to how they are managed (Parliamentary Commissioner for the Environment 2018).

Firstly, we considered all the NGMP sites together in a single group (Figure 4A). Linear regression produces a slope that is significantly different from zero (*p* < 0.01). The regression line has an intercept of 1.42 ± 0.53 mg/l, which is interpreted to be the 80th percentile for NO_3_–N concentration in groundwater in the absence of any NO_3_–N leached from livestock (Table 1).

We performed a second evaluation in which the NGMP sites were segregated into hydrochemical facies, and each site’s weighting in the regression was scaled according to its fraction of groundwater aged 5 years or less (Figure 4B). This grouping of the NGMP sites according to their hydrochemical facies helps to understand any observed relationships between NO_3_–N leaching rate and the observed NO_3_–N concentrations in groundwater, and the age-weighting maximises the regression influence for those sites with residence time distributions most closely corresponding to the 2017 year used for the NO_3_–N leaching map. Note that sites assigned to Cluster 2 are anoxic and hence denitrification is likely to have removed almost all NO_3_–N from the groundwater, which explains the lack of discernible relationship to mapped NO_3_–N leached from livestock. Sites assigned to Clusters 1A and 1B are oxic, so are more likely to show relationships between NO_3_–N concentration and the mapped NO_3_–N leached from livestock. Based on their hydrochemistry, sites assigned to Cluster 1A are inferred to show evidence of human/agricultural impact and so, following the approach of McDowell et al. (2013), the 80th percentile for NO_3_–N concentration in minimally impacted groundwater can be inferred from the intercept of the regression line (1.87 ± 1.63 mg/l). By comparison, sites assigned to Cluster 1B are interpreted to have little or no evidence of human impact. The Cluster 1B regression line has an intercept of 0.99 ± 0.45 mg/l and a slope that is not significantly different from zero (*p* > 0.1). In other words, even though NO_3_–N leaching from livestock is mapped in the vicinity of some Cluster 1B sites, the impacts of this leaching are not observable as elevated NO_3_–N concentrations in the groundwater. Given that Cluster 1B groundwaters are predominantly oxic and hence would have low likelihood for the occurrence of denitrification, we suggest that a lack of relationship between NO_3_–N concentration in the groundwater and mapped NO_3_–N leaching from livestock could indicate that these sites are recharged predominantly from river seepage rather than rainfall passage through the soil zone, and/or recharged from an area outside the 2.0 km radius used in this study. In either case, the similarity of the intercepts for the Cluster 1A and Cluster 1B regression lines provides independent but very similar estimates for the 80th percentile for NO_3_–N concentration in oxic, minimally impacted groundwater in New Zealand. Finally, the regression line for Cluster 2 has an intercept of 0.05 ± 0.45 mg/l and a slope that is not significantly different from zero (*p* > 0.1), providing an estimate of the 80th percentile NO_3_–N concentration that would be expected for anoxic groundwaters. Similar approaches can be used to derive estimates for other percentiles of NO_3_–N concentration in oxic and anoxic groundwater under reference conditions (Table 1).

We repeated the analyses described above using the LAWA dataset. Again, using the 80th percentile as an example, linear regression based on all LAWA sites as a single group (Figure 4C) yielded an intercept of 2.23 ± 0.43 mg/l (Table 1), providing a similar estimate as derived from the NGMP dataset for of the 80th percentile for NO_3_–N concentration in groundwater in the absence of any NO_3_–N leached from livestock. As noted above, including all the LAWA sites in the regression does not account for differences in the main hydrochemical drivers such as redox condition or potential degree of human impact. We therefore developed separate regression lines by categorising the LAWA sites according to their hydrochemical facies as based on the LDA model (Figure 4D). LPM parameters are not presently compiled for the LAWA sites, so it is not possible to weight each site’s influence in the regressions according to its groundwater age distribution. For the 80th percentile, the resulting regression for Cluster 1A sites yields an intercept of 6.73 ± 0.77 mg/l, which is markedly different from the result obtained with the same method applied to the NGMP sites (1.87 ± 1.63) and indeed from all other methods and datasets employed in this study (Table 1). Further, some LAWA sites assigned to Cluster 1B have markedly elevated NO_3_–N concentrations. We conclude that the uncertainties of the LDA model (potentially causing some sites to be assigned to the incorrect cluster) and the lack of groundwater age information hampers the utility of this approach when applied to the LAWA dataset.

We identify certain advantages and limitations of estimating reference conditions for groundwater quality based on regression against a measure of land-use impact. One clear advantage is that land-use impacts are quantified for each site, whereas the other methods employed in this study can only infer the extent of land-use impacts from indirect proxies. A challenge is to account for each site’s capture zone, groundwater transit time, and any hydrochemical processes, such as denitrification, any of which could confound the relationship between NO_3_–N leaching rates and the observed concentrations of NO_3_–N in groundwater. As shown in this study, these challenges can be reasonably overcome through combined use of a circular area surrogate for the capture zone, LPMs to describe groundwater age distribution, and HCA to assign sites to distinct hydrochemical facies.

### Reference conditions for NO_3_–N in New Zealand groundwater

#### Results obtained from different methods, datasets and time periods

The previous sections show that there are strengths and weaknesses for each of the three methods used to estimate reference conditions in this study. When applied to a single dataset and time period, the three methods generally produce very similar estimates for the selected percentiles in NO_3_–N concentrations expected for oxic, minimally impacted groundwater in New Zealand (Table 1). We conclude that there is no clear reason to favour one method over another, and so results from all three methods should be included in the weight of evidence approach for estimation of NO_3_–N concentrations under reference conditions. The case of estimating reference conditions for anoxic groundwater is discussed later in this section.

The previous sections also show that all three methods can be applied to the NGMP dataset, whereas there are limitations for the application of any of the three methods to the LAWA dataset. For the HCA method, the LAWA dataset lacks most of the analytical parameters so requires an LDA model to predict cluster assignments, which introduces uncertainty to the estimation of reference conditions. The LAWA dataset lacks the required information for the groundwater age method. For the method of regression against a measure of land-use impact, the results from the LAWA dataset suggest a much higher NO_3_–N concentration in oxic minimally impacted groundwater than is estimated from any other methods and datasets employed in this study. Aside from these limitations associated with application of each method, the LAWA dataset may also contain a systematic bias because many of the sites are selected to monitor at-risk aquifers where elevated NO_3_–N concentrations are already known to exist (Daughney et al. 2012). We therefore conclude that results from the LAWA dataset should not be used in the weight of evidence approach, but instead should only be used for validation and cross-comparison of the results obtained from the NGMP dataset.

The previous sections also show that very similar results are obtained for the two periods (2000–2009 and 2010–2019) for most methods and datasets (Table 1). This finding is to be expected because groundwater chemistry is relatively static or otherwise changing quite slowly at most long-term monitoring sites in New Zealand (Moreau and Daughney 2021). In turn this suggests that the natural processes that influence NO_3_–N concentrations under reference conditions are relatively steady over the two ten-year periods used in this study. Natural processes that could influence NO_3_–N concentrations in groundwater could include the El Niño-Southern Oscillation climate pattern (Fleming and Quilty 2006; Snelder et al. 2021), which could affect volumes of groundwater recharge or discharge, temperature, or fluxes of atmospheric nitrogen deposition, any of which could influence groundwater NO_3_–N concentrations. We therefore conclude that results from both ten-year time periods should be included in the weight of evidence approach.

Based on the discussion above, we estimated reference conditions for oxic groundwater using results from the three different methods and both ten-year time periods but applied only to the NGMP dataset (Table 2). By this approach, the weighted averages and corresponding 95% confidence intervals for the 50th, 80th, 90th and 95th percentiles of NO_3_–N in oxic, minimally impacted groundwater were found to be 0.76 ± 0.09, 1.65 ± 0.12, 1.97 ± 0.14 and 2.32 ± 0.14, respectively.

**Table 2.** Weighted averages for selected percentiles ± 95% confidence intervals for NO_3_–N concentration (mg/l) in oxic, minimally impacted groundwater as determined in this study using the highlighted results from Table 1.

Percentile	NO_3_–N Concentration
5th	0.14 ± 0.03
10th	0.17 ± 0.03
20th	0.31 ± 0.05
50th	0.76 ± 0.09
80th	1.65 ± 0.12
90th	1.97 ± 0.14
95th	2.32 ± 0.14

Anoxic groundwaters can also exist under natural conditions but tend to have NO_3_–N concentrations that are near or below the analytical detection limit. As such, this study treats anoxic groundwaters as a separate population with its own definition of reference conditions (Daughney and Reeves 2005). This study has shown that only some of the methods are suitable for anoxic groundwater, but across both periods these yielded weighted average estimates of the 80th and 90th percentiles of NO_3_–N of 0.04 ± 0.01 and 0.16 ± 0.01, respectively (results not tabulated). The 95th percentile for NO_3_–N in anoxic groundwater could not be reliably estimated from the methods used in this study.

Overall, the strong similarity in the estimates of reference condition derived from the different methods, time periods and datasets suggest that methodological approaches introduced relatively little bias. We acknowledge that the replacement of censored values with their corresponding detection limits may have affected the application of certain methods to certain sites, particularly those sites with very low NO_3_–N concentrations. For example, the observed shift in the proportion of sites assigned to Cluster 1 vs Cluster 2 between the two time periods (Figure 2) may partially reflect a methodological artefact arising from the treatment of censored values. However, the definition of reference conditions, particularly for the upper percentiles in NO_3_–N, are much more dependent on the apportioning of sites between Cluster 1A and Cluster 1B, which is observed to be constant between the two time periods.

We caution against using the results from this study to estimate a single definition of reference conditions that combines the expected NO_3_–N concentration in both oxic and anoxic groundwater. First, this study has been unable to reliably estimate the 5th, 10th, 20th, 50th and 95th percentiles for NO_3_–N in anoxic groundwater, so these results are not available for merging with the equivalent percentiles for oxic groundwater. Second, the methods used in this study can determine whether groundwater is anoxic, but not whether it is unequivocally unimpacted by human activity (while NO_3_–N is a clear marker for human impact in oxic groundwater in New Zealand, the typical low concentrations in anoxic groundwater environments does not necessarily indicate a lack of human impact). Third and most importantly, to estimate a single distribution of NO_3_–N concentration for all unimpacted groundwaters would require knowledge of the proportion of oxic versus anoxic groundwater across the entire country, which to date has only been estimated for selected depths (Wilson et al. 2020), not volumetrically across all aquifer systems.

#### Comparison to previous studies

The results from this study are in good general agreement with previously reported thresholds for NO_3_–N in groundwater under reference conditions (Table 3). For example, European guidance suggests a 90th percentile of 2.26 mg/l NO_3_–N (10 mg/l as NO_3_) (Pauwels et al. 2007), which is similar to the value of 1.97 ± 0.14 derived in this study for minimally impacted oxic groundwater in New Zealand. Other studies listed in Table 3 have reported 90th percentile thresholds in the range from 1.08 to 3.25 mg/l NO_3_–N, and 95th percentile thresholds in the range from 0.56 to 3.5 mg/l.

**Table 3.** Comparison of results from this study (grey shading) to examples of previously reported thresholds for NO_3_–N concentration in groundwater under reference conditions.

NO_3_–N Threshold		Location	Notes	Methods	Reference
Concentration	Threshold type				
0.2	N.R.	New Zealand (national scale)	Oxic groundwaters only	1	Morgenstern and Daughney (2012)
0.56	95th percentile	Mountainous area of Jeju Island, South Korea		2	Koh et al. (2009)
∼1	N.R.	England and Wales	Upland areas only	2	Shand et al. (2007)
1 to 3	N.R.	Europe		2	Shand and Edmunds (2008)
1.08 to 1.74	90th percentile	Banat Groundwater Body, Romania		1, 3	Radu et al. (2010)
1.13	N.R.	West Bank, Palestine		N.R.	Anayah and Almasri (2009)
1.24	95th percentile	Mountainous area of Jeju Island, South Korea		2	Koh et al. (2009)
1.44	95th percentile	Chalk Aquifer, United Kingdom		4	Limbrick (2003)
1.6	75th percentile	New Zealand (national scale)	Oxic groundwaters only	5	Daughney and Reeves (2005)
1.65 ± 0.12	80th percentile	New Zealand (national scale)	Oxic groundwaters only	1, 5, 6	This study
1.85	90th percentile	Tarim River Basin, Northern China		1	Huo et al. (2012)
1.97 ± 0.14	90th percentile	New Zealand (national scale)	Oxic groundwaters only	1, 5, 6	This study
∼2	N.R.	Minqin Basin, Northwest China		1	Edmunds et al. (2006)
2.17	90th percentile	Loess Plateau, Northern China		1	Huo et al. (2012)
2.26	90th percentile	Europe		1, 3	Pauwels et al. (2007)
2.32 ± 0.14	95th percentile	New Zealand (national scale)	Oxic groundwaters only	1, 5, 6	This study
3	N.R.	United States (national scale)		N.R.	Madison and Brunett (1985)
∼3 to 4	N.R.	England and Wales	Non-upland areas only	2	Shand et al. (2007)
3.25	90th percentile	North China Plain, Northern China		1	Huang et al. (2012)
3.5	95th percentile	New Zealand (national scale)	Oxic groundwaters only	5	Daughney and Reeves (2005)
3.9	90th percentile	Bono, Ahafo and Bono East regions, Ghana		7, 8	Manu et al. (2022)
∼5	N.R.	Upper Pantanoso Stream Basin, Argentina		N.R.	Costa et al. (2002)
6.7	N.R.	Chalk Aquifer, United Kingdom	Confined parts of aquifer only	2	Edmunds et al. (2003)
7.5	N.R.	Chalk Aquifer, United Kingdom	Unconfined parts of aquifer only	2	Edmunds et al. (2003)

Rows are ordered according to increasing NO_3_–N threshold concentration. N.R. = Not reported. Methods are: 1 = Comparison to groundwater age; 2 = Cumulative probability distribution; 3 = Selection methods; 4 = Assessment of historical data; 5 = Hierarchical cluster analysis; 6 = Regression against measure of land-use impact; 7 = Iterative outlier removal; 8 = Gaussian mixture model.

There are three factors that complicate a more detailed comparison between results from this study and the earlier work (see Table 3). First, several of the previous studies did not specify the percentile corresponding to their reported threshold. For example, human influence on groundwater has been inferred for NO_3_–N concentrations above 3 mg/l in the United States (Madison and Brunett 1985) or above 3–4 mg/l in England and Wales (Shand et al. 2007), but these studies did not state whether their reported thresholds correspond to the 80th, 90th, 95th percentile or some other metric. Second, most previous studies have not reported the confidence intervals or uncertainty of their reported thresholds. A related issue is that most previous studies have used only one method for the estimation of reference conditions. As a result, across previous studies it is not straightforward to compare the different estimates for any single threshold from a perspective of statistical significance. Third, from some of the previous studies it is not clear whether the reported thresholds were derived from oxic and anoxic groundwaters separately, as in this study, or together. This study has shown that there can be considerable variation in groundwater chemistry depending on aquifer redox status, groundwater ages, and land management, which means that determination of a robust and representative reference conditions should ideally be based on more than one method, particularly to assess whether there is cause for different definitions of reference conditions for different aquifer types, lithologies or redox statuses. Despite these challenges, we conclude that the percentiles derived in this study for NO_3_–N in oxic, minimally impacted groundwater in New Zealand are generally similar to previous estimates from many other parts of the world.

It is also instructive to compare this study’s national-scale estimates for reference conditions to previous local-scale investigations of NO_3_–N concentration in New Zealand aquifers that were inferred to be minimally impacted. We caution that such comparisons must be made with care because, as noted in Introduction section, previous groundwater investigations in pristine catchments are limited in number, not necessarily designed to infer thresholds for minimally impacted conditions, and do not yet provide representative coverage of all important groundwater settings in New Zealand. This said, some previous local-scale investigations in New Zealand have reported minimally impacted oxic groundwaters with NO_3_–N concentrations falling in the lower half of the range of reference conditions as derived in this study. Such previous site- and catchment-scale investigations include but are not limited to the fractured marble aquifer system of Te Waikoropupū Spring (0.3–0.46 mg/l, Moreau 2021) and selected sites in the pumice and ignimbrite aquifers of the Lake Taupo catchment (0.1–0.3 mg/l, Morgenstern 2008; Morgenstern and Daughney 2012), the ignimbrite and rhyolite aquifers of the Lake Roturua catchment (0.4–0.7 mg/l, Morgenstern et al. 2004), and some parts of the gravel aquifers of the Heretaunga Plains (0.1–0.5 mg/l, Morgenstern et al. 2018). These lower NO_3_–N concentrations may reflect settings with nitrate leaching rates at the lower end expected under certain types of native forest (0–2.5 kg N per ha per year, Davis 2014). These lower NO_3_–N concentrations may also indicate some degree of denitrification, which is possible even in aquifer conditions that appear to be oxic overall (Utom et al. 2020). Denitrification in oxic aquifers has been noted in New Zealand (Martindale et al. 2019) and could be accentuated in several of the local-scale studies cited in this paragraph which had groundwaters with mean age of >50 or even >100 years.

Likewise, some previous local-scale investigations in New Zealand have reported minimally impacted oxic groundwaters with NO_3_–N concentrations falling in the upper half of the range of reference conditions as derived in this study. We acknowledge that in some cases the slightly elevated NO_3_–N concentrations may reflect a low level of human impact. However, some pristine groundwaters in the Lake Rotorua and Lake Taupo catchments can have NO_3_–N concentrations from 0.8 to 1.8 mg/l (Morgenstern et al. 2004; Morgenstern 2008), which due to geothermal activity may experience increased nitrate leaching rates even in areas of native vegetation (Davis 2014), as well as inflows of nitrogen from the subsurface. Groundwater NO_3_–N concentrations of ca. 2 mg/l are also observed in the Takaka limestone aquifer (Stevens 2010), a concentration commensurate with overseas studies suggesting that nitrification of organic-N may occur within such karstic systems (Angel and Peterson 2015; Musgrove et al. 2016), and potentially other lithologies as well (Utom et al. 2020). Aside from geothermal influence or nitrification of organic-N, other natural processes that could potentially contribute to comparable NO_3_–N concentrations (ca. 2 mg/l) include relatively high leaching losses in areas of native nitrogen-fixing plants (Dollery et al. 2019), or concentration of NO_3_–N due to groundwater evapotranspiration. Further investigations are required to determine whether these processes are important in New Zealand groundwater systems.

#### Use of estimates of reference conditions for groundwater management

Individual jurisdictions make the policy choice of whether an estimate of reference conditions should be used in groundwater management. For example, in Europe, under the Water Framework Directive (2000/60/CE) and its daughter directive, the Groundwater Directive (2006/118/EC), information on reference conditions is used as a basis to develop and implement river basin management plans (De Stefano et al. 2013; Urresti-Estala et al. 2013). By comparison, in New Zealand, use of reference conditions for groundwater management is not specifically mandated, but the National Policy Statement for Freshwater Management does define ‘attribute bands’ for certain parameters in surface water that in some cases include thresholds that are representative of minimally disturbed conditions (New Zealand Ministry for the Environment 2022). Regional authorities in New Zealand can implement groundwater management objectives and thresholds more stringent than required through national policy, but to date there has been little application of reference conditions for NO_3_–N for this purpose in regional policies or plans.

Where a choice is made to manage groundwater based on reference conditions, individual jurisdictions must select the threshold to be applied. For example, for implementation of the Groundwater Directive, the 90th percentile is recommended as an appropriate threshold for defining reference conditions for groundwater quality where location-specific studies have not determined any other threshold as more appropriate (Pauwels et al. 2007). In New Zealand, the 20th and 80th percentiles are used as thresholds to define Default Guideline Values for surface water quality, but no analogous thresholds have yet been included for groundwater quality (ANZG 2018).

We propose that the 80th percentile is an appropriate default threshold for characterisation of reference conditions in New Zealand groundwater, given that this same default threshold is already applied for surface waters. Note that such a default guideline value is not necessarily a management limit or target that needs to be met; rather, exceedance may simply be intended to serve as a prompt to consider whether further investigation should be undertaken to determine whether aquatic ecosystems are sufficiently protected from human impacts (see ANZG 2018). Note also that the 80th percentile is simply recommended as a default threshold, allowing water management entities to undertake investigations and define different percentile-based thresholds for specific aquifers or parts of aquifers, depending on the level of protection deemed necessary.

Once a percentile-based threshold has been selected for reference conditions, it can be compared against measured concentrations when reporting on the state of the environment. Ideally, comparison to the reference conditions should also take account of uncertainty in the value of whatever percentile-based threshold used (e.g. threshold + confidence limit). We suggest that applications in environmental reporting should aim to clearly convey that a proportion of monitoring sites is expected to exceed the threshold even under minimal human impacts; for example, 20% of monitoring sites will have median NO_3_–N concentrations higher than the 80th percentile identified for reference conditions. We also caution that, in our experience, precision in language is needed for effective use of descriptions of estimated reference conditions in state of the environment reporting. We suggest wording such as ‘there is 95% confidence that 80% of oxic minimally impacted groundwaters in New Zealand have 10-year median NO_3_–N concentrations equal to or less than 1.77 mg/l’. While this wording is a bit cumbersome, it clearly indicates the selected percentile plus its upper 95% confidence limit, explains that the relevant monitoring metric is a long-term median, and conveys that the conclusion pertains only to oxic, minimally impacted groundwaters.

## Conclusions

This study has illustrated that reference conditions for NO_3_–N in groundwater are helpfully estimated through the application of a range of complementary methods, datasets, and monitoring time periods. At the national scale in New Zealand, we estimate that the 80th percentile in NO_3_–N concentration is 1.65 ± 0.12 for oxic, minimally impacted groundwater and 0.04 ± 0.01 for anoxic groundwater, using data collected through the National Groundwater Monitoring programme over the period 1 January 2000 to 31 December 2019. In keeping with the approach used for New Zealand surface waters (ANZG 2018), we suggest that the 80th percentile should be used as national default threshold for reference conditions, for comparison to the NO_3_–N concentrations observed in environmental monitoring programmes, except where site-specific investigations have indicated that different threshold values should be used. While this study has focussed on NO_3_–N, the same approaches could be used for estimation of reference conditions for other groundwater quality variables.

## Supplementary Material

Supplemental material

## Data Availability

Groundwater quality data from the NGMP are available from GNS Science at https://ggw.gns.cri.nz. Groundwater age interpretations for the NGMP sites are provided in the Supplementary Material for this publication. Groundwater quality data from the LAWA data are available from www.lawa.org.nz.
